# Community Structure Reveals Biologically Functional Modules in MEF2C Transcriptional Regulatory Network

**DOI:** 10.3389/fphys.2016.00184

**Published:** 2016-05-24

**Authors:** Sergio A. Alcalá-Corona, Tadeo E. Velázquez-Caldelas, Jesús Espinal-Enríquez, Enrique Hernández-Lemus

**Affiliations:** ^1^Computational Genomics Department, National Institute of Genomic MedicineMexico City, Mexico; ^2^Complexity in Systems Biology, Center for Complexity Sciences, Universidad Nacional Autónoma de MéxicoMexico City, Mexico

**Keywords:** community structure, transcriptional regulatory networks, MEF2C, FANTOM4, InfoMap, transcription factor

## Abstract

Gene regulatory networks are useful to understand the activity behind the complex mechanisms in transcriptional regulation. A main goal in contemporary biology is using such networks to understand the systemic regulation of gene expression. In this work, we carried out a systematic study of a transcriptional regulatory network derived from a comprehensive selection of all potential transcription factor interactions downstream from MEF2C, a human transcription factor master regulator. By analyzing the connectivity structure of such network, we were able to find different biologically functional processes and specific biochemical pathways statistically enriched in communities of genes into the network, such processes are related to cell signaling, cell cycle and metabolism. In this way we further support the hypothesis that structural properties of biological networks encode an important part of their functional behavior in eukaryotic cells.

## Introduction

### Transcriptional gene regulation and systems biology

Gene expression is the phenomenon in which a series of coupled biochemical interactions will produce all needed proteins for a specific cell. Some of these physical-chemical processes involve DNA, RNA and also a number of proteins generically called **transcription factors** (TFs). They work together in order to synthesize (or transcribe) mRNA from which, eventually, will produce a protein. TFs are then proteins that are able to bind specific sites of DNA (promoter sites) thus allowing (or preventing) mRNA synthesis by attaching to DNA into specific genomic regions [The transcription factor binding site (TFBS)].

The particular structure of the relationships between TFs and their targets determines a given phenotype (Ravasi et al., 2010). This structure-function coupling, however, is not easy to be observed experimentally. In order to grasp the strongly linked relationship between structure and function of transcriptional regulation, a Systems Biology-based approach is thus necessary. In this regard, **network theory** has emerged as a major tool to achieve this phenomenon (Barabási et al., 2000; Newman, 2010). In our case, it is possible to model the transcriptional regulation through a complex network, where the genes are represented by nodes in the network, and links correspond to the physico-chemical constraints or conditions in gene regulation, these networks are commonly called Gene Regulatory Networks (GNRs; Thattai and Van Oudenaarden, 2001; Davidson and Levin, 2005; Davidson and Erwin, 2006; Olson, 2006).

### Network theory and community detection

Complex networks are theoretical models useful to represent the integrated behavior of interacting agents in a vast number of scenarios in nature and society. Often, global organization patterns of large complex networks involve the presence of structural sub-units (subnetworks) that have been called *modules* or *communities*. There is not a clear cut consensus of what is the key element that made such modules distinctive entities within the network. However, a generally accepted notion is that communities within a network correspond to tightly interconnected sets of vertices such that the density of *within-connections* is higher than that of *between-connections* (Girvan and Newman, 2002; Clauset et al., 2004; Palla et al., 2005; Newman, 2006; Rosvall and Bergstrom, 2007, 2008; Fortunato, 2010).

Community detection in networks, is still an open problem in computer science (Mucha et al., 2010) and there are a huge variety of detection methods and algorithms (Gulbahce and Lehmann, 2008; Ahn et al., 2010; Fortunato, 2010; Xie et al., 2013); thus, community structure is an issue of particular relevance in the case of gene transcriptional regulatory networks (Tang et al., 2012), where modules or communities correspond to co-regulated sets of genes (Wilkinson and Huberman, 2004; Zhu et al., 2008; Marbach et al., 2012; Cantini et al., 2015).

Here, we considered particularly the case of MEF2C that is a transcription factor (TF) gene associated with muscle growth and developmental processes (Baca-López et al., 2012), and encodes a 473-amino acid protein. Furthermore, it is a member of the *Mef2* family that controls gene expression and it is able to regulate cellular differentiation and development (Potthoff and Olson, 2007). MEF2 molecules are highly versatile regulators commonly acting as activators of transcription factors. Their protein-protein interactions, mostly with other transcription factors, enhancers or epigenomic regulators, together with its inherent binding-site transcriptional activity render MEF2C a functional and adaptable master regulator (MR; Hernández-Lemus et al., 2015).

### Outline

**Figure 1:**
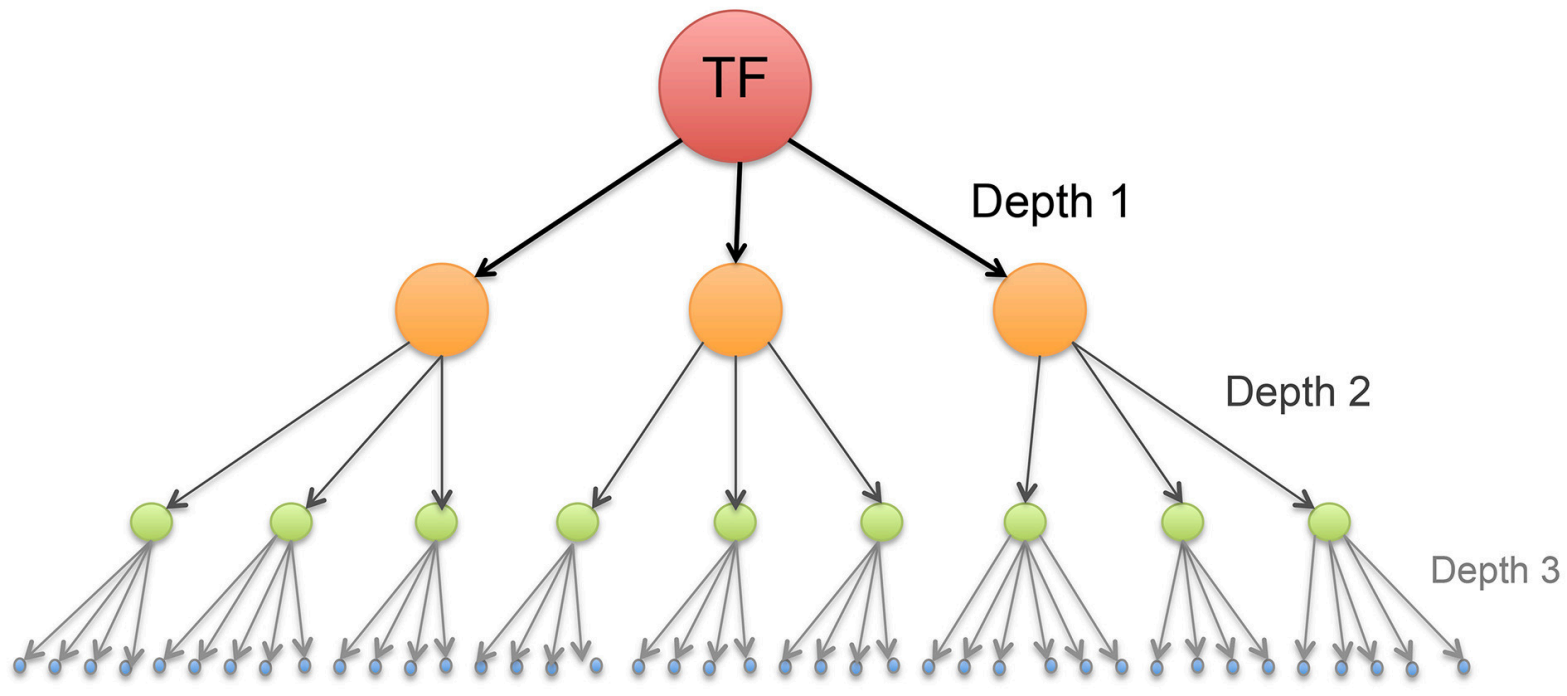
**Schematic representation of a transcription network composed by a master regulator gene (like the MEF2C Transcription Factor) and its targets down to depth = 3**. The directions of the arrows point to target gene.

Our purpose in this work, hence, is to study the modular connectivity structure in a transcriptional regulatory network, and to analyze whether those communities are involved in specific biological processes. This is done by inferring a network from *transcription factor binding sites* (TFBS) –namely the network consisting in all the downstream transcriptional targets (down to three-layers) of the MEF2C molecule– (Figure 1), some of these targets may even be TF of some molecules in upstream layers resulting in the presence of cycles. Once modules or communities were revealed, we analyzed the presence of characteristic biological functions in specific communities, to look up for functional module behavior.

The rest of the article goes as follows: Next section will present the main analytical methods and models used to investigate the community structure in this transcriptional regulatory network: namely, the TFBS network construction; the implementation of **infoMap** community detection algorithm, and the enrichment analysis to find biological relevance for each detected community. The results section contains the main findings of our methodology. The inferred communities reveal that specific sets of genes participate in particular biological processes. A null model validates the community structure of the MEF2C transcriptional network. Finally, we discuss the implications that this link between structure and functionality have in biological networks.

## Methods and models

**Figure 2:**
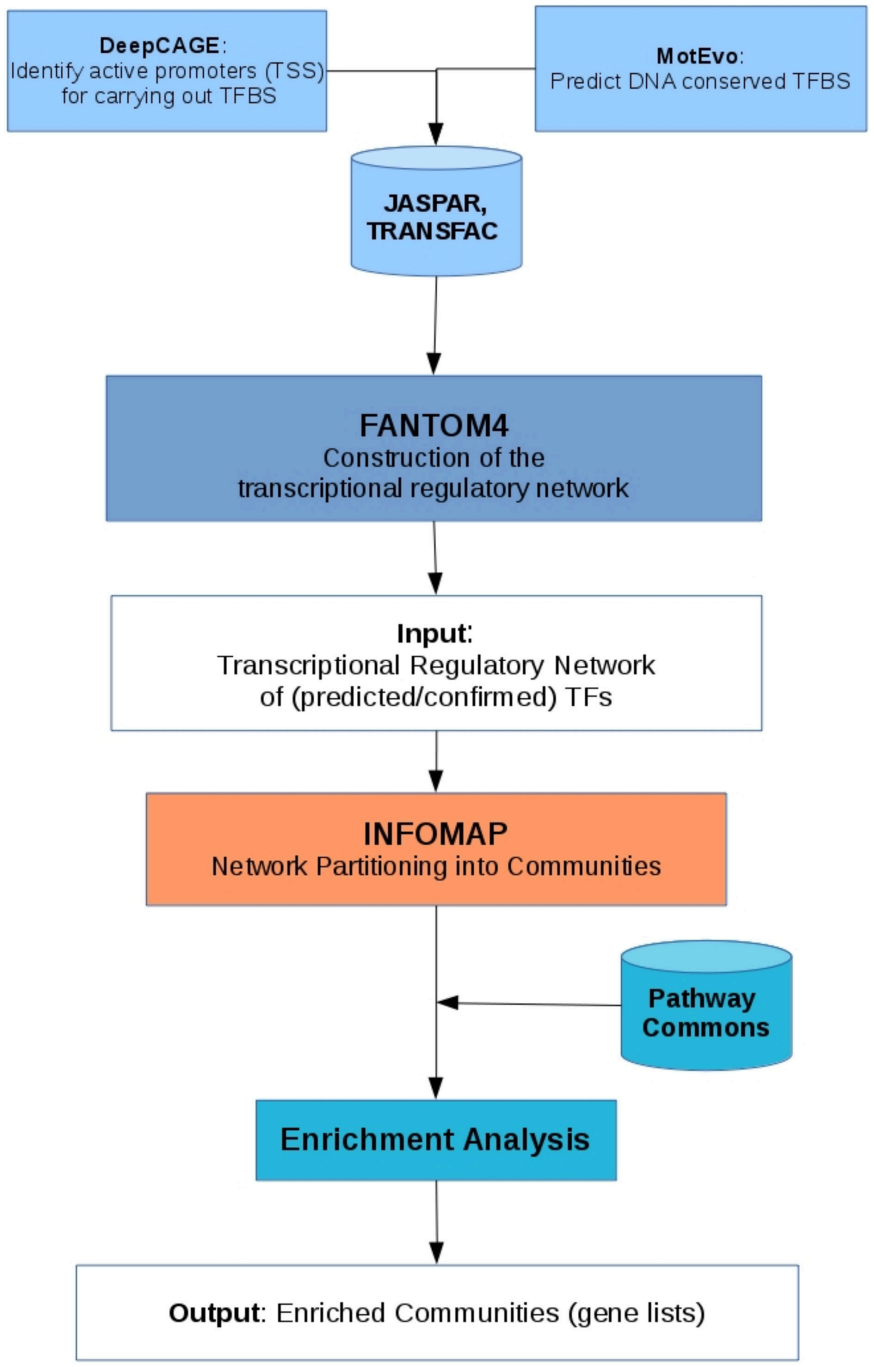
**Chart diagram of the methodology used, that describe the inferring of the network, the community detection and the enrichment analysis**.

The methodology used for the present study can be summarized in three very general parts, first, the inference of the MEF2C transcriptional regulatory network, then the partition of the network into modules using the InfoMap algorithm and finally, perform an enrichment analysis of the communities found. Below we shall detail each of these steps. A summary of this methodology is shown in Figure 2.

### Transcription factor binding site (TFBS) analysis and network construction.

In order to infer this transcriptional regulatory network, we consider all the downstream transcriptional targets of the MEF2C molecule (Figure 1) by employing the FANTOM4 database^1^ whose experimental procedure is explained below.

First, using deep sequencing techniques (deepCAGE) to monitor transcription starting sites (TSS) at a single-base-pair level of resolution, it is possible to identify active promoters and define relevant regions for carrying out Transcription Factor Binding Site (TFBS) predictions. On the other hand, to infer patterns of DNA conserved TFBS, the MotEvo algorithm is used (Arnold et al., 2012). This is a Bayesian framework to integrate multiple DNA sequence alignments used to infer transcriptional regulation interactions.

Using both methods, FANTOM4 can infer that a gene *X* is a TF of *Y* gene, if the promoter associated with a TSS-motif in *Y* has a predicted regulatory TFBS-motif in *X*, and the edge between them has independent experimental support (Suzuki et al., 2009). With this database, we build the MEF2C network up to three levels, i.e., the targets of MEF2C (first level); the targets of those targets (second level), and the targets of the latter (third level). It is worth to mention that some of the first, second and third level targets may be in turn regulators of the upper levels, thus indicating the presence of loops in this transcriptional regulatory network.

The experimental support for such high throughput technique was performed using databases like JASPAR^2^ or TRANSFAC^3^ which provides a curated, non-redundant set of profiles, derived from published collections of experimentally defined transcription factor binding sites for eukaryotes, their experimentally-proven binding sites, consensus binding sequences (positional weight matrices) and regulated genes.

Given the aforementioned, FANTOM4 is a reliable source of TFBS information to build a network model of gene expression based on transcription factors and its regulation.

### Community structure detection analysis

Community structure is relevant for the large scale structure of complex biological networks (Gulbahce and Lehmann, 2008). It would be desirable to identify groups of proteins or genes that are connected in the network in order to establish relationships between these groups and a biological function, and even be able to make predictions of the unknown function of some genes, based on the module or community they belong to. Thus, the prediction of functions associated with gene groups is one of the most promising results derived from applying graph partition and clustering techniques. This will help us to improve our understanding of the underlying relationship between structure and function in biological networks.

In order to discover such pattern of local and midscale interconnectivity (in particular in directed, weighted networks), several methodologies have been proposed (Fortunato, 2010), and some of them have been successfully applied to the case of Protein-Protein Interaction Networks (*PPI networks*) where the modules are consistent with groups of proteins associated with biological functions (Rives and Galitski, 2003; Spirin and Mirny, 2003; Chen and Yuan, 2006; Farutin et al., 2006; Jonsson et al., 2006; Lewis et al., 2010).

One of such methodologies is a theoretical approach based on compressing the information needed to describe the process of *information diffusion* across a network. The foregoing is proxied by a random walk. This approach implemented in a method called infomap (Rosvall and Bergstrom, 2008), leads to a natural decomposition of the network into modules or communities. In addition to the theoretical background and precise application of those ideas, we have chosen the Infomap algorithm, since it has proven to be highly efficient compared to other methods. Based on benchmarks, Infomap was the best-ranked method in runtime, accuracy and performance. For a detailed description of the method, please see reference (Rosvall and Bergstrom, 2008; Lancichinetti and Fortunato, 2009).

The general notion is that groups of nodes constituting a community (a single, well connected module) will be those for which information flows in a quick, easy manner as compared to nodes *outside-of-the-community*. In terms of a random walk, groups of nodes highly interconnected are communities, hence it is intuitive that a walker will spend more time within them. Similar to geographic maps, where much information can be encoded in a few structures within it (for instance, a line represents a river), and that the lacking of the large detail of how it is, does not affect their interpretation of the map; the method use Huffman coding (Huffman, 1952) to name nodes, and gives unique names (*codewords*) to structures in the network that play a crucial role in the process of *information diffusion*. Every time the walker “jumps” to a different module (structure coded in the network), one needs to use the codeword of that community in the description, to inform the decoder of the transition. The foregoing is done by updating the codewords of each cluster until we reach a final partition of the graph.

Within this coding approach, an important question arises: *How can we choose a code that allows the optimal description of paths on a given network arising from a random walk process in a way that it reflects the underlying structure of the network?* The answer as shown in reference (Rosvall and Bergstrom, 2008), is a clever application of Shannon's source coding theorem (Huffman, 1952; Shannon and Weaver, 1959). The description length (the length of codeword) consists of two terms, expressing the Shannon entropy of the random walk within and between modules. Thus, finding the actual community structure in networks is akin to find an optimal coding. To perform it, one starts by looking for a *partition* M consisting of *n* nodes distributed according to the coding rule into *m* modules in a way that minimizes the expected description length of a random walk.

If community structure is well defined, the transitions of the random walker between modules will be sporadic, and in the description of the random walk, the codewords of the modules will not be repeated many times. On the other hand, if there are no well-defined modules, or the partition is not representative of the current community structure of the network, transitions between the clusters of the partition will be very frequent, and the description length of the modules will be large. Thereby, minimization of the description length, and therefore finding the best partition M is carried out by combining greedy search with simulated annealing.

**Figure 3:**
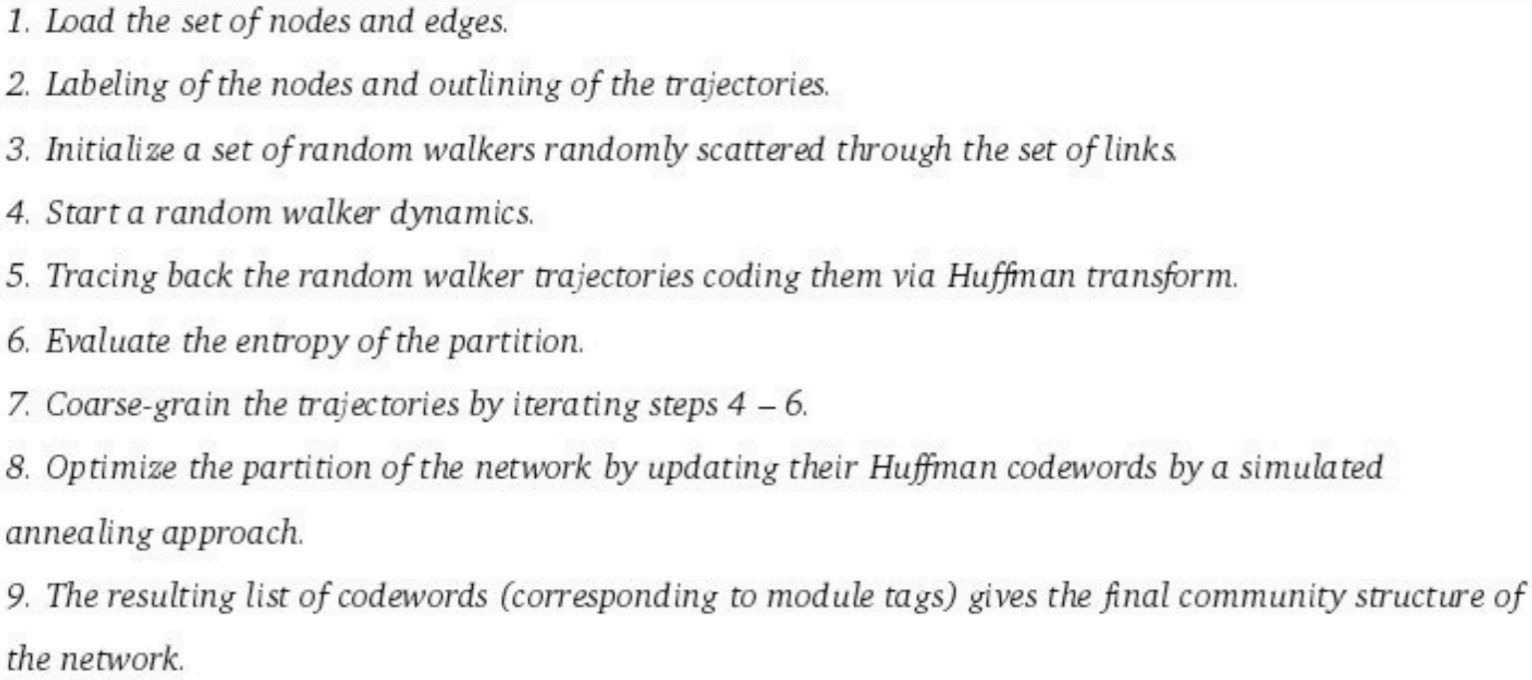
**Heuristic description of the infomap algorithm**.

Thus, identifying modules and finding community structure within a network is tantamount to solving a coding problem, i.e., to find the partition that yields the minimum description length (in terms of codewords) of an infinite random walk (Rosvall and Bergstrom, 2007). Just like a good geographic map encodes the important information about a place, Infomap maps the whole system-wide information flow, thus retaining the weights and directions of the links. Those are relevant if it is desired to know not only the connectivity, but also the information flow patterns in the network. This results in stark contrast with traditional methods, that often disregard both (weights and directions) placing a somehow excessive emphasis in the local *degree* and *centrality* distributions. For a better understanding of the algorithm we have included a box with the heuristic description of the algorithm (Figure 3).

#### Null model construction

In order to validate the aforementioned results, we constructed a null model in which all the nodes of the MEF2C network were conserved as well as the number of links, but the links were randomly rewired according to the Erdös-Rényi model (Erdös and Rényi, 1959), in which any pair of nodes taken at random on the network is connected with a probability *p*. The previous procedure approximates the connections of the nodes to a binomial distribution which tends to a Poisson distribution if the number of nodes is large.

### Enrichment analysis

A relevant goal of large scale molecular studies of biological systems is to reach a well-founded description of the functional processes associated with phenotypic cell functioning (Hernández-Lemus, 2013). Systems level studies are focusing more and more on reaching an integrative mechanistic or semi-mechanistic understanding of cellular systems. An approach that has proved to be quite useful in this regard is the one called *enrichment analysis* (Zhang et al., 2005; Wang et al., 2013; García-Campos et al., 2015).

There are several ways to perform enrichment analysis, but the general rationale is approximately the same: if a relatively-large number of molecules associated with a particular biological process are present in the system, there is a probability that such process is *active*. To specify what is a *relatively-large number* we need to know how many molecules are present with respect to those that are representative of the processes and compare this with a null model to assess statistical significance. The common way to do this is by means of hypergeometric tests, usually corrected due to multiple hypothesis testing.

#### Hypergeometric tests

Statistical enrichment analysis in this context, relies on the determination of statistical over/under-representation of annotated biochemical pathways within a given network or set of genes. Significance assessment may be performed by means of so-called *urn model*-based hypergeometric distribution tests. Testing a given biological pathway amounts to drawing the genes annotated at it from the *urn* (our gene universe) and classifying them as to whether they belong to such pathway by means of an indicator function. By counting all possible queries and calculating the resulting proportions, it is possible to perform a hypergeometric test of statistical significance (that in the present case is equivalent to a one-tailed Fisher's Exact test).

#### False discovery rate

In order to correct for multiple testing, Benjamini-Hochberg algorithm correction –the so called False Discovery rate (FDR)– can be used. Only associations whose corrected *p*-values ranged below a pre-established threshold, were considered significant. Pathway enrichment analysis in this work, was performed by mapping common biologically functional pathways for a given gene set on the full Pathway Commons database, and considering the output of this mapping as a graph.

## Results

On what follows, we will present the main results of this study, namely: (i) the TFBS inferred MEF2C transcriptional network and its main topological features, (ii) the network community substructure, (iii) the statistically significant biological functional features found in the communities and, (iv) validation of the significance of all these findings by contrast with a null model.

### Transcriptional network for MEF2C targets is implied in complex phenotypes

**Figure 4:**
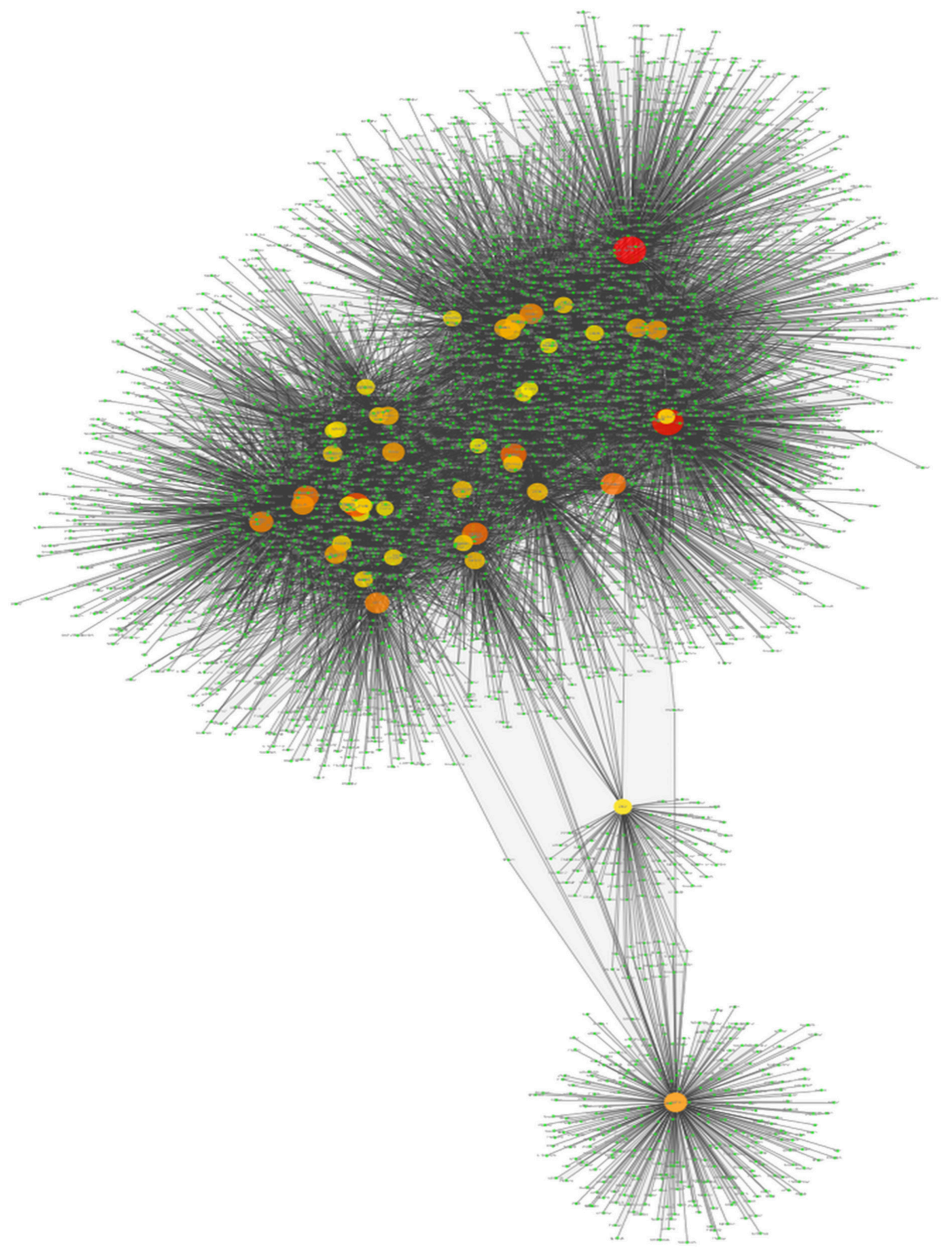
**Transcriptional network of TFBS interactions for the MEF2C transcription factors and its targets up to the third level**. In this visualization, the color and the size of the genes is depicted according to node-degree (number of neighbors connected to this particular gene): small green nodes correspond to barely-connected genes; whereas larger orange and red nodes represent highly connected genes.

The inferred MEF2C transcription factor network (Figure 4) is composed of 4543 nodes and 12,422 links (see Table 1). This network shows some highly connected genes (such as GABPA, ELK4, CREB1, MYC, or MEF2C), represented by bigger circles. For instance, MEF2C is deeply implied in muscle growth and development (Sartorelli et al., 1997). The topological analysis of this network is presented in Table 1. It is remarkable that MEF2C, HMGA2 and others belong to a group of highly relevant TFs known as Master Regulators, which corroborate previous results regarding cancer phenotypes (Potthoff and Olson, 2007; Baca-López et al., 2012).

**Table 1:** **Main Topological parameters of the MEF2C transcriptional network**.

Clustering coefficient	0.250	Network diameter	5
Network centralization	0.208	Characteristic path length	3.170
Average number of neighbors	5.440	Network heterogeneity	6.288
Number of vertices	4543	Number of edges	12422
Node degree distribution	*P*(*k*) = 278.27 × *k*^−1.044^	Correlation degree dist.	0.952
Clustering coefficient distribution	*C*(*k*) = 1.907 × *k*^−1.042^	Correlation clustering dist.	0.860

### Community structure reveals subnetworks related to specific biological processes

#### Community network topology

**Figure 5:**
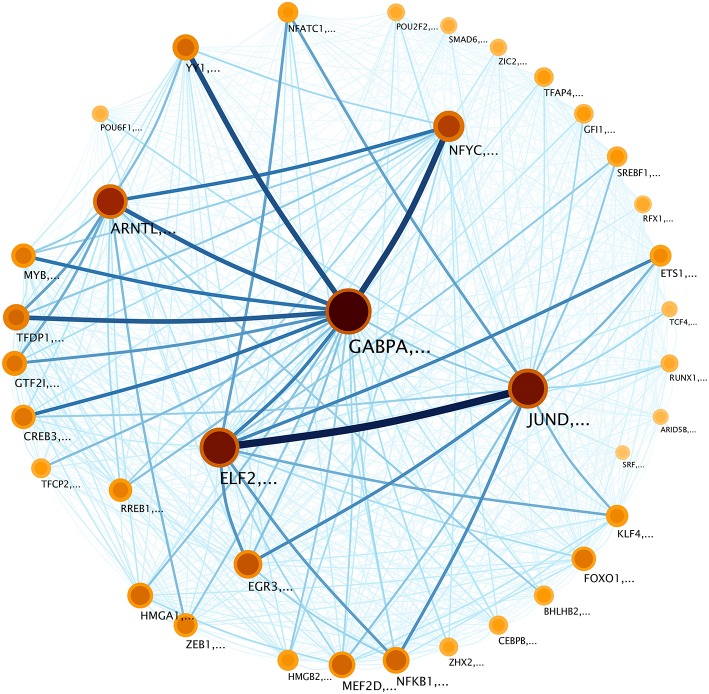
**Community structure of the transcriptional network in Figure 4**. Modules are tagged with the name of the node whit the highest PageRank (Brin and Page, 2012) inside the community. Nodes (which represent communities) are depicted according to the size of the community and the information flow inside that community. In this sense, darker colors correspond to larger information contents whereas bigger circles represent larger communities. The relative degree of information flow is depicted in the width and color of the inter-module links. The thickness of the module borders reflects the probability that a random surfer within the module, will follow a regulation (edge) to a gene outside of the module. The weighted links between communities represent *regulation flow*, with the color and width of the edges indicating flow volume. For example, the lines between JUND and ELF2 communities, indicate information flow of regulation between them. These links reveal the relationship between communities.

The infomap network visualization (Figure 5) contains information about the communities appearing in the network. These communities vary in the number of molecules and the flow of information that the communities share among them (For lists of genes for each community see Supplementary Data Sheet 1). For instance, in this network, GABPA, ELF2, and NFYC tagged-communities are the largest and the most sharing networks, in terms of information (Figure 5). It is worth to mention that circles represent communities (in a coarse-grained perspective) that contain themselves several nodes. The name of the communities is given by the node with the highest Page-rank score (Brin and Page, 2012). The Page-rank score works by counting the number and quality (its Page-rank itself) of links to a node to determine a rough estimate of how important the node is.

To clarify Figure 5, let us explain the graphical aspects of it as it was originally performed in Rosvall and Bergstrom (2008). The size of each module or “gene-community” on the map reflects the fraction of time that a random surfer [a random walker with a “teleportation probability” to “jump” to another random node in the network (Rosvall and Bergstrom, 2008)] spends following gene interactions within that module.

As it can be observed, the size of each community varies, however, the time spent for the random surfer within a community is not always proportional to the size of that community. For instance, the community associated with GABPA gene includes 643 genes and the aforementioned random surfer spends 14% of its time within this community: This is the reason for which the node GABPA is the biggest one in Figure 5. On the other hand, NFYC community includes 309 genes, however, the random surfer spends 5.1% of its time into this community. The remaining question after this analysis is whether those communities have a specific role in biological processes, e.g., if they participate as a multi-gene-complex during particular events in the cell.

#### Functional communities participate in different biological processes

**Figure 6:**
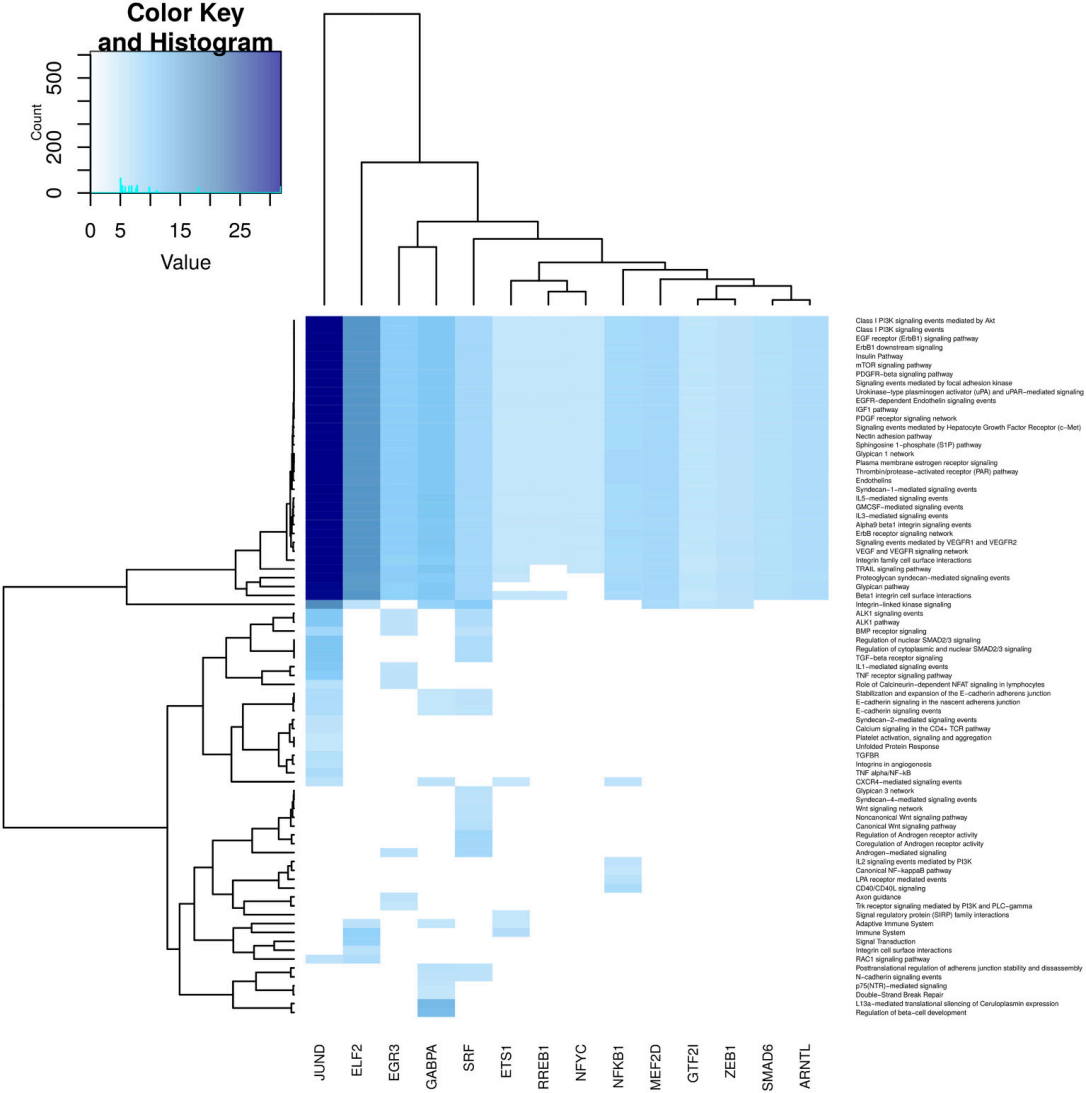
**Heatmap depicting enrichment in pathways related to *cell signaling* processes**. Communities are tagged with the name of its higher PageRank molecule. Color intensity is proportional to the −*log* of *p*-value. Darker spots correspond to statistically significant hits. The upper-left inset shows the Z-score histogram and color key for the *p*-value of the enriched processes. Finally, a dendrogram which shows similar *p*-value distributions among the enriched processes in the communities is also depicted.

**Figure 7:**
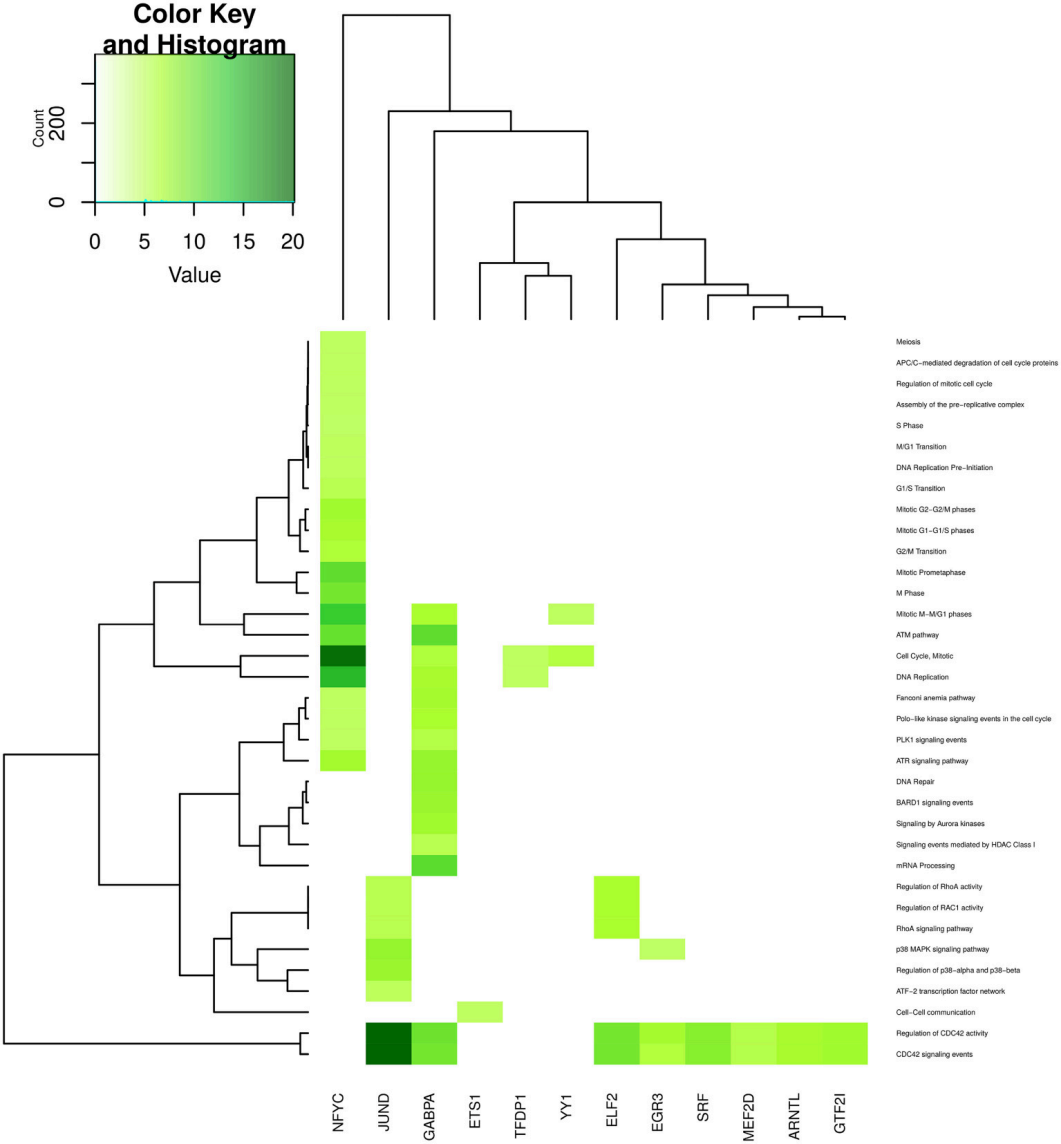
**Heatmap depicting enrichment in pathways related to *cell cycle***. Communities are tagged with the name of its higher PageRank molecule. Color intensity is proportional to the −*log* of *p*-value. Darker spots correspond to statistically significant hits. The upper-left inset shows the Z-score histogram and color key for the *p*-value of the enriched processes. Finally, a dendrogram which shows similar *p*-value distributions among the enriched processes in the communities is also depicted.

**Figure 8:**
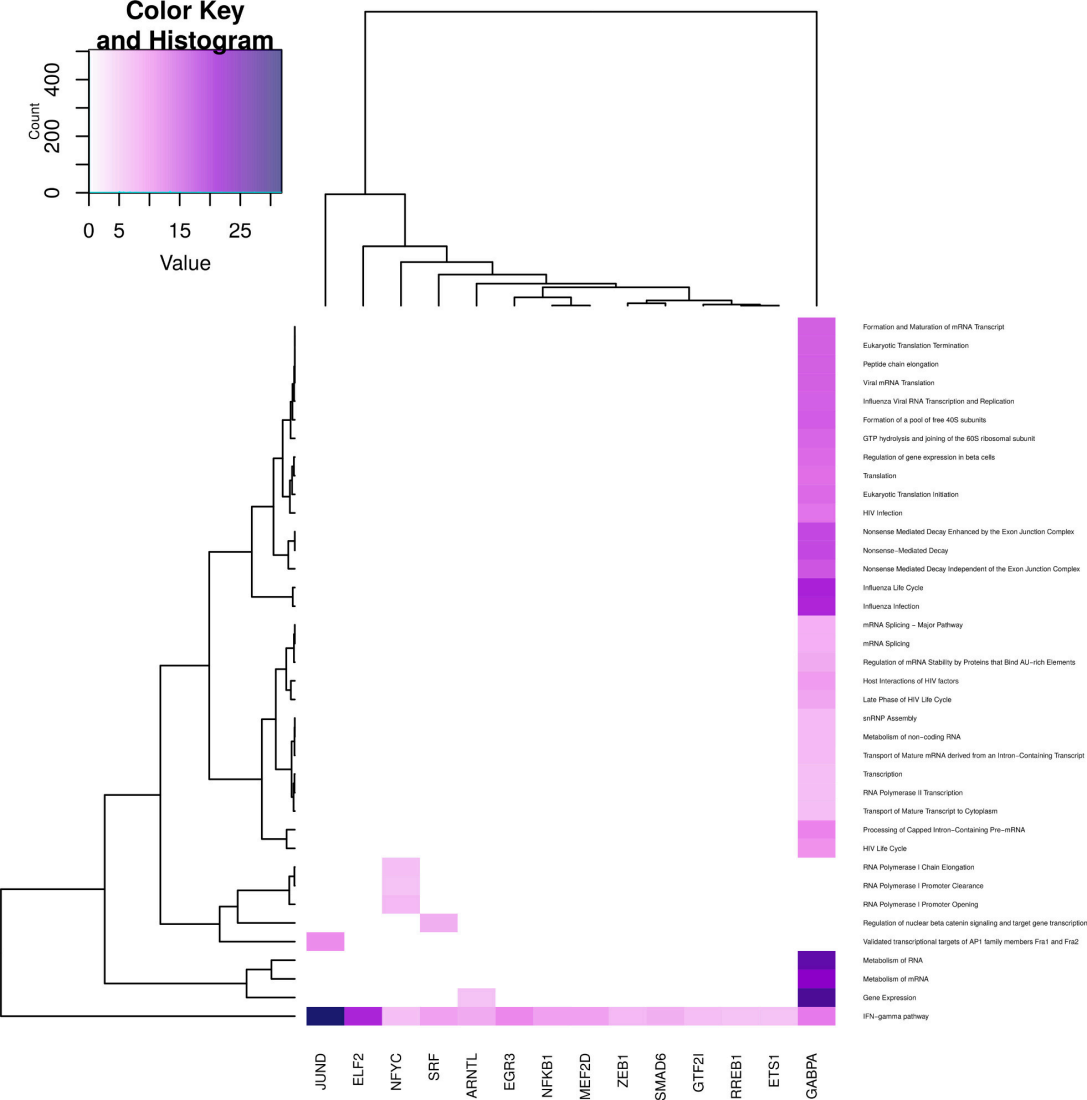
**Heatmap depicting enrichment in pathways related to *gene expression***. Communities are tagged with the name of its higher PageRank molecule. Color intensity is proportional to the −*log* of *p*-value. Darker spots correspond to statistically significant hits. The upper-left inset shows the Z-score histogram and color key for the *p*-value of the enriched processes. Finally, a dendrogram which shows similar *p*-value distributions among the enriched processes in the communities is also depicted.

**Figure 9:**
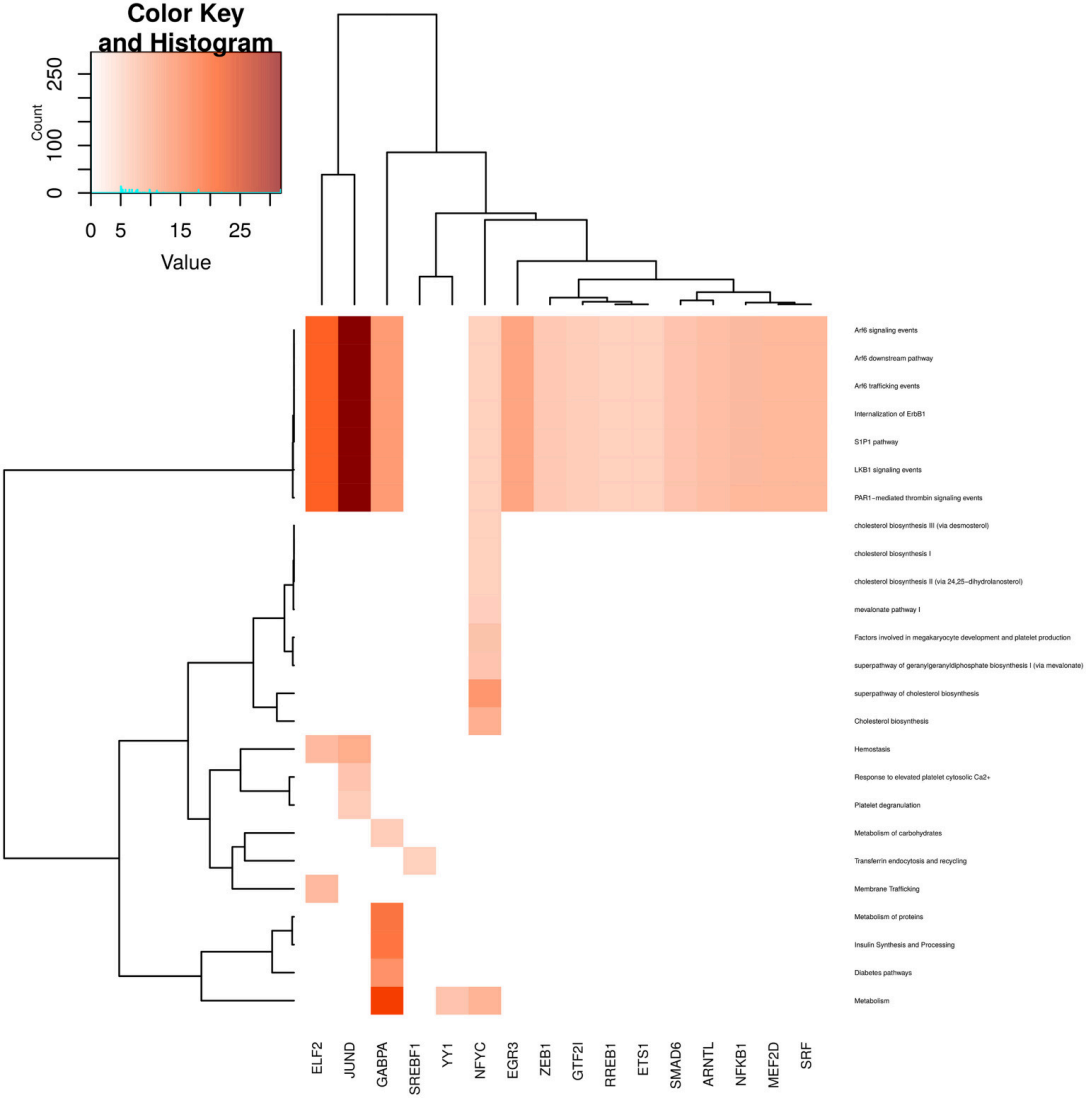
**Heatmap depicting enrichment in pathways related to *metabolism and cellular transport processes***. Communities are tagged with the name of its higher PageRank molecule. Color intensity is proportional to the −*log* of *p*-value. Darker spots correspond to statistically significant hits. The upper-left inset shows the Z-score histogram and color key for the *p*-value of the enriched processes. Finally, a dendrogram which shows similar *p*-value distributions among the enriched processes in the communities is also depicted.

In Figures 6–**9**, we can see heatmaps of FDR-corrected *p*-values showing statistically significant enrichment of biological pathways within specific communities in the network (Supplementary Data Sheet 2). Color intensity is proportional (Z-score) to −log_10_[*p*−*value*]. Figures 6–**9**, represent the processes which are enriched in communities related to cell signaling (6), cell cycle (7), gene expression (8) and metabolism (9).

Regarding Figure 6, which refers to cell signaling processes, a strong enrichment in JUND and ELF2 communities can be observed. It is also highlighted the fact that several processes are enriched in all communities (upper part of the heatmap). More specific processes are enriched in GABPA, EGR3 and ETS1 communities. We can notice that processes related to the immune system are also enriched in NFKB community, reinforcing the fact that NFKB gene participates in immune system responses. Finally, it is worth to mention that many cell signaling-related processes are broadly enriched in the SRF community, despite it is relatively small (17 genes).

In the case of Figure 7 (unlike Figure 6) for processes related to cell cycle and DNA structure, less than a quarter of these are enriched in the JUND-tagged community; however, the most enriched processes of this category belong to JUND community -**regulation of CDC42 activity** (*p* = 7.04 × 10^−21^) and **CDC42 signaling events** (*p* = 1.21 × 10^−20^), respectively-. Processes such as **assembly and degradation of cell cycle proteins** are only enriched in the NFYC community, meanwhile **DNA repair and mRNA processing** are enriched exclusively in GABPA community. Again, a very specific process like **cell-cell communication** is only enriched in ETS1 community.

Regarding the gene expression processes (Figure 8), all categories, but those related to **transcription** are enriched in GABPA community; instead, transcription events are enriched in NFYC community. This is another instance of the specificity regarding the functionality of the topology-inferred communities. On the other hand, **IFN-**γ **pathway** is enriched in all communities (bottom part of the figure), reflecting the relevance of this process. It is also worth to mention that **regulation of nuclear** β **catenin signaling and target gene transcription** process again is enriched only in the SRF community.

By looking at Figure 9 (metabolic processes), similar to signaling-related processes, a subset of those processes are enriched in almost all communities. These are more related to **Arf6 signaling** and internalization metabolic processes. These processes are highly enriched in JUND community. Moreover, diabetes-related events and protein metabolism are enriched in GABPA community. More specific events, such as **Transferrin endocytosis and recycling** are only enriched in the SREBF1 community; NFYC community is specific to 8 processes, mainly related to cholesterol metabolism and platelet production and finally, membrane trafficking is specifically enriched in ELF2 community.

We can see that the modules are associated with specific biological processes, as given by their respective pathways, i.e., such pathways are enriched in that community. In some cases processes are uniquely associated with a specific community, whereas in another, a given biological process may be enriched in several modules.

#### MEF2C transcriptional network community structure is validated by a null model

**Figure 10:**
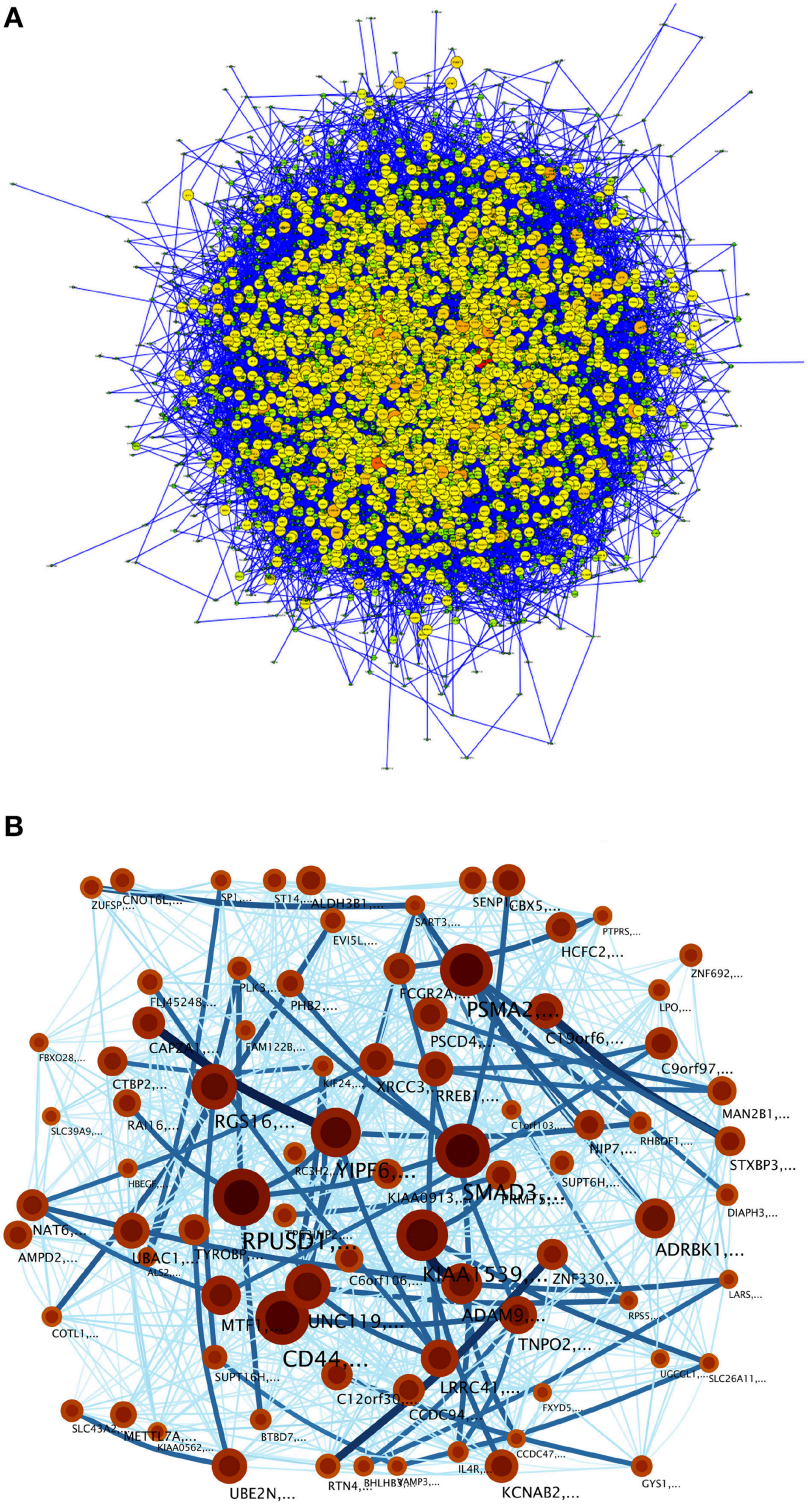
**(A)** A null model constructed by the Transcriptional network for TFBS interactions for the MEF2C transcription factors and its targets up to the third level into an Erdös-Renyí network with the same nodes and the number of edges but randomized links. **(B)** Community structure of the null model. Modules are tagged as explained in Figure 5.

Figure 10 shows the null-model-network (NMN) topology (Figure 10A) and the infomap network (Figure 10B) where no community structure was found, since partitions of the network corresponded to just a few genes. Thus, none pathway commons category resulted significantly enriched (at the *p* < 10^−5^ level) in any of the NMN communities.

## Discussion

In this work we have studied the community structure of a transcriptional network, associated to a relevant transcription factor MEF2C. We have found that the community structure of the Fantom4-curated network contains biologically meaningful information. By analyzing the set of highly-connected genes, we observed that these have a relevant role in the network structure with biological implications (as we will discuss on what follows):

### Genes related to specific communities have relevant roles in gene regulation

*GABPA* is a transcription factor which contains a DNA-binding motif. It is involved in activation of cytochrome oxidase expression and nuclear control of mitochondrial function (Vaughan et al., 2014). Interestingly enough, *GABPA* is a well-known transcription factor which belongs to the ETS family, and it regulates different target genes associated with cytoskeletal functions and cell migration control (Odrowaz and Sharrocks, 2012).

*NFYC*: According to the literature, this gene is a highly conserved transcription factor that is activator of a variety of genes. This transcription factor is related to DNA repairing (Tong et al., 2015), regulation of transcription (Lützner et al., 2012) and its malfunction is associated with development of different types of carcinomas (Kottorou et al., 2012).

On the other hand, *JUND*, an intronless gene, is a member of the JUN family, and a functional component of the AP1 transcription factor complex, and it has been proposed to protect cells from p53-dependent senescence and apoptosis. This gene is one of the most important cancer-related genes (Gazon et al., 2012; Huang et al., 2012; Nakayama et al., 2012; Eckhoff et al., 2013; Li et al., 2013; Thevenon et al., 2013; Davidson et al., 2014; Wang et al., 2014; Zou et al., 2015), but also is relevant in apoptotic events and DNA repair (Zerbini et al., 2011), proliferation (Caffarel et al., 2008) and oxidative stress (Mehraein-Ghomi et al., 2008).

Finally, *SRF* transcription factor encodes an ubiquitous nuclear protein (Taylor and Halene, 2015) related to cell proliferation (Liu et al., 2014) and differentiation (Liao et al., 2015). Its protein binds to the serum response element (SRE) in the promoter region of target genes and regulates the activity of many immediate-early genes, such as c-fos, and thereby participates in cell cycle regulation, apoptosis and Epithelium-mesenchymal transition (Bae et al., 2014). It is worth to mention that the SRF community is the smallest one with enriched processes according to our criterion threshold (*p* < 10^−5^); nevertheless, specific cell signaling-related processes are highly enriched in this community. More research regarding the functionality of this community is mandatory.

### Structure and function are strongly related to the MEF2C transcriptional networkce

Community structure analysis, in turn, reveals that there are specialized communities in one particular process (i.e., such process is strongly enriched in those subnetworks), the foregoing suggest that the co-expression of this set of genes (a community) may be directly associated with such cellular process in an *adaptive* way. In addition, there are several processes that are enriched in more than one community (see the columns corresponding to JUND-tagged community in Figures 6–9), in this case it is possible to argue that these communities collaborate together giving *robustness* to the system in terms of gene sets.

A quite interesting result obtained by the community inference analysis is that in each of the biggest communities, the information flow is controlled by Transcription Factors (GABPA, NFYC, JUND, etc.), and they are in turn regulated by MEF2C which reinforces our statement regarding the fact that MEF2C is a Transcriptional Master Regulator. These last results lead us to argue that the structure of the community is strongly linked to the functionality in the MEF2C transcription network.

It is important to note that community structure is related not only with the structural and functional partitioning of the network, in the sense of communities performing individual functions (as is shown in Figures 6–9 and the related discussion). As a close inspection of Figure 5 reveals, the regulation information flow between communities implies cross-community regulatory patterns. Hence, communities may be functioning like *pieces of machinery* involved in the processes of several subsystems conforming a device. At the local level they perform highly specialized actions that in the context of the global performance of the machine also collaborate with other subsystems on the device.

It is worth to mention that the infomap algorithm has barely been used to detect communities in biological networks (Sethi et al., 2009; Komurov et al., 2010; Power et al., 2011). However, this algorithm has been proven to be more efficient and faster than other more used for networks with biological meaning (Fortunato, 2010; Cantini et al., 2015). Due to this, we decided to use infomap algorithm to infer the existing communities in the MEF2C network.

The community detection algorithm as well as the enrichment analysis performed here can be applied to a large variety of networks, which can be inferred with different approaches. This constitutes an important feature of the methodology presented here. Enrichment analysis of detected communities, may provide insights to grasp the complexity underlying the transcription regulation process.

### Summary

Here we present an analysis of the transcription factor binding sites network of MEF2C gene. We decomposed the network into communities by means of the infomap algorithm. The communities obtained here, are highly specialized in particular cellular processes, for instance, the communities tagged by GABPA and JUND.

With the methodology performed here, we were able to discover functional communities related to specific biological processes. This is another instance in which structure reveals functionality. Interestingly enough, the fact that MEF2C is disposed at the third level of regulation also implies that there are looped mechanisms of regulation intrinsic to the structure of the network. Further investigation on this issue is needed to understand the concrete mechanisms. Notwithstanding, this work is a first approach to answer such a fundamental question.

## Author contributions

SA: Performed analyses and calculations, outlined results, collaborated in writing of the manuscript. TV: Contributed to biological analyses, collaborated in the outline of results, read and approved the manuscript. JE: Performed analyses, collaborated in the outline of results, collaborated in writing the manuscript. EH: Designed the study, performed analyses and calculations, collaborated in writing the manuscript.

## Funding

This work was supported by CONACYT (grant no.179431/2012), as well as by federal funding from the National Institute of Genomic Medicine (Mexico). Additional support has been granted by the National Laboratory of Complexity Sciences (grant no. 232647/2014 CONACYT). Sergio Antonio Alcalá-Corona is a doctoral student from Programa de Doctorado en Ciencias Biomédicas, Universidad Nacional Autónoma de México (UNAM) and received fellowship 391657 from CONACYT.

### Conflict of interest statement

The authors declare that the research was conducted in the absence of any commercial or financial relationships that could be construed as a potential conflict of interest.

## Footnotes

^1^http://fantom.gsc.riken.jp/4/edgeexpress/view

^2^http://jaspar.genereg.net/

^3^http://www.gene-regulation.com/pub/databases.html

## Supplementary material

Supplementary Data Sheet 1 **The communities obtained by the methodology performed here**. This tab-separated file contains the whole set of genes involved in each community.

Supplementary Data Sheet 2 **Table files containing the statistically significant enriched processes in specific communities in the network**. The processes are separated as is explained in the article: cell cycle, cell signaling, gene expression and metabolism.

## References

[B1] Ahn Y.-Y. Bagrow J. P. Lehmann S. (2010). Link communities reveal multiscale complexity in networks. Nature 466, 761–764. 10.1038/nature09182 20562860

[B2] Arnold P. Erb I. Pachkov M. Molina N. van Nimwegen E. (2012). MotEvo: integrated Bayesian probabilistic methods for inferring regulatory sites and motifs on multiple alignments of DNA sequences. Bioinformatics 28, 487–494. 10.1093/bioinformatics/btr695 22334039

[B3] Baca-López K. Mayorga M. Hidalgo-Miranda A. Gutiérrez-Nájera N. Hernández-Lemus E. (2012). The role of master regulators in the metabolic/transcriptional coupling in breast carcinomas. PLoS ONE 7:e42678. 10.1371/journal.pone.0042678 22952604 PMC3428335

[B4] Bae J. S. Noh S. J. Kim K. M. Jang K. Y. Chung M. J. Kim D. G.. (2014). Serum response factor induces epithelial to mesenchymal transition with resistance to sorafenib in hepatocellular carcinoma. Int. J. Oncol. 44, 129–136. 10.3892/ijo.2013.2154 24173109

[B5] Barabási A.-L. Albert R. Jeong H. (2000). Scale-free characteristics of random networks: the topology of the world-wide web. Physica A 281, 69–77. 10.1016/S0378-4371(00)00018-2

[B6] Brin S. Page L. (2012). Reprint of: the anatomy of a large-scale hypertextual web search engine. Comput. Netw. 56, 3825–3833. 10.1016/j.comnet.2012.10.007

[B7] Caffarel M. Moreno-Bueno G. Cerutti C. Palacios J. Guzman M. Mechta-Grigoriou F.. (2008). JunD is involved in the antiproliferative effect of Δ9-tetrahydrocannabinol on human breast cancer cells. Oncogene 27, 5033–5044. 10.1038/onc.2008.145 18454173

[B8] Cantini L. Medico E. Fortunato S. Caselle M. (2015). Detection of gene communities in multi-networks reveals cancer drivers. Sci. Rep. 5:17386. 10.1038/srep17386 26639632 PMC4671005

[B9] Chen J. Yuan B. (2006). Detecting functional modules in the yeast protein–protein interaction network. Bioinformatics 22, 2283–2290. 10.1093/bioinformatics/btl370 16837529

[B10] Clauset A. Newman M. E. Moore C. (2004). Finding community structure in very large networks. Phys. Rev. E 70:066111. 10.1103/PhysRevE.70.066111 15697438

[B11] Davidson C. L. Cameron L. E. Burshtyn D. N. (2014). The AP-1 transcription factor JunD activates the leukocyte immunoglobulin-like receptor 1 distal promoter. Int. Immunol. 26, 21–33. 10.1093/intimm/dxt038 24038602

[B12] Davidson E. Levin M. (2005). Gene regulatory networks. Proc. Natl. Acad. Sci. U.S.A. 102, 4935–4935. 10.1016/j.biosystems.2010.08.003 15809445 PMC556010

[B13] Davidson E. H. Erwin D. H. (2006). Gene regulatory networks and the evolution of animal body plans. Science 311, 796–800. 10.1126/science.1113832 16469913

[B14] Eckhoff K. Flurschütz R. Trillsch F. Mahner S. Jänicke F. Milde-Langosch K. (2013). The prognostic significance of Jun transcription factors in ovarian cancer. J. Cancer Res. Clin. Oncol. 139, 1673–1680. 10.1007/s00432-013-1489-y 23942796 PMC11824582

[B15] Erdös P. Rényi A. (1959). On random graphs. Public. Math. Debrecen 6, 290–297. 20808879

[B16] Farutin V. Robison K. Lightcap E. Dancik V. Ruttenberg A. Letovsky S.. (2006). Edge-count probabilities for the identification of local protein communities and their organization. Proteins 62, 800–818. 10.1002/prot.20799 16372355

[B17] Fortunato S. (2010). Community detection in graphs. Phys. Rep. 486, 75–174. 10.1016/j.physrep.2009.11.002

[B18] García-Campos M. A. Espinal-Enríquez J. Hernández-Lemus E. (2015). Pathway analysis: state of the art. Front. Physiol. 6:383. 10.3389/fphys.2015.00383 26733877 PMC4681784

[B19] Gazon H. Lemasson I. Polakowski N. Césaire R. Matsuoka M. Barbeau B.. (2012). Human T-cell leukemia virus type 1 (HTLV-1) bZIP factor requires cellular transcription factor JunD to upregulate HTLV-1 antisense transcription from the 3 long terminal repeat. J. Virol. 86, 9070–9078. 10.1128/JVI.00661-12 22696638 PMC3416116

[B20] Girvan M. Newman M. E. (2002). Community structure in social and biological networks. Proc. Natl. Acad. Sci. U.S.A. 99, 7821–7826. 10.1073/pnas.122653799 12060727 PMC122977

[B21] Gulbahce N. Lehmann S. (2008). The art of community detection. BioEssays 30, 934–938. 10.1002/bies.20820 18800363

[B22] Hernández-Lemus E. (2013). Further steps toward functional systems biology of cancer. Front. Physiol. 4:256. 10.3389/fphys.2013.00256 24065926 PMC3776302

[B23] Hernández-Lemus E. Baca-López K. Tovar H. (2015). What makes a transcriptional master regulator? A systems biology approach, in Physical Biology of Proteins and Peptides, eds Olivares-Quiroz L. Guzmán-López O. Jardón-Valadez H. E. (Springer), 161–174. 10.1007/978-3-319-21687-4_10

[B24] Huang J. Gurung B. Wan B. Matkar S. Veniaminova N. A. Wan K.. (2012). The same pocket in menin binds both MLL and JUND but has opposite effects on transcription. Nature 482, 542–546. 10.1038/nature10806 22327296 PMC3983792

[B25] Huffman D. A. (1952). A method for the construction of minimum-redundancy codes. Proc. IRE 40, 1098–1101. 10.1109/JRPROC.1952.273898

[B26] Jonsson P. F. Cavanna T. Zicha D. Bates P. A. (2006). Cluster analysis of networks generated through homology: automatic identification of important protein communities involved in cancer metastasis. BMC Bioinformatics 7:2. 10.1186/1471-2105-7-2 16398927 PMC1363365

[B27] Komurov K. White M. A. Ram P. T. (2010). Use of data-biased random walks on graphs for the retrieval of context-specific networks from genomic data. PLoS Comput. Biol. 6:e1000889. 10.1371/journal.pcbi.1000889 20808879 PMC2924243

[B28] Kottorou A. E. Antonacopoulou A. G. Dimitrakopoulos F.-I. D. Tsamandas A. C. Scopa C. D. Petsas T.. (2012). Altered expression of NFY-C and RORA in colorectal adenocarcinomas. Acta Histochem. 114, 553–561. 10.1016/j.acthis.2011.10.005 22104449

[B29] Lancichinetti A. Fortunato S. (2009). Community detection algorithms: a comparative analysis. Phys. Rev. E 80:056117. 10.1103/PhysRevE.80.056117 20365053

[B30] Lewis A. C. Jones N. S. Porter M. A. Deane C. M. (2010). The function of communities in protein interaction networks at multiple scales. BMC Syst. Biol. 4:100. 10.1186/1752-0509-4-100 20649971 PMC2917431

[B31] Li C. Li S. Kong D.-H. Meng X. Zong Z.-H. Liu B.-Q.. (2013). BAG3 is upregulated by c-Jun and stabilizes JunD. Biochim. Biophys. Acta 1833, 3346–3354. 10.1016/j.bbamcr.2013.10.007 24140207

[B32] Liao X.-H. Wang N. Zhao D.-W. Zheng D.-L. Zheng L. Xing W.-J.. (2015). STAT3 protein regulates vascular smooth muscle cell phenotypic switch by interaction with myocardin. J. Biol. Chem. 290, 19641–19652. 10.1074/jbc.M114.630111 26100622 PMC4528129

[B33] Liu Z. Zhang J. Gao Y. Pei L. Zhou J. Gu L.. (2014). Large-scale characterization of DNA methylation changes in human gastric carcinomas with and without metastasis. Clin. Cancer Res. 20, 4598–4612. 10.1158/1078-0432.CCR-13-3380 25009298 PMC4309661

[B34] Lützner N. Arce J. D.-C. Rösl F. (2012). Gene expression of the tumour suppressor LKB1 is mediated by Sp1, NF-Y and FOXO transcription factors. PLoS ONE 7:e32590. 10.1371/journal.pone.0032590 22412893 PMC3295762

[B35] Marbach D. Costello J. C. Küffner R. Vega N. M. Prill R. J. Camacho D. M.. (2012). Wisdom of crowds for robust gene network inference. Nat. Methods 9, 796–804. 10.1038/nmeth.2016 22796662 PMC3512113

[B36] Mehraein-Ghomi F. Lee E. Church D. R. Thompson T. A. Basu H. S. Wilding G. (2008). JunD mediates androgen-induced oxidative stress in androgen dependent LNCaP human prostate cancer cells. Prostate 68, 924–934. 10.1002/pros.20737 18386285

[B37] Mucha P. J. Richardson T. Macon K. Porter M. A. Onnela J.-P. (2010). Community structure in time-dependent, multiscale, and multiplex networks. science 328, 876–878. 10.1126/science.1184819 20466926

[B38] Nakayama T. Higuchi T. Oiso N. Kawada A. Yoshie O. (2012). Expression and function of FRA2/JUND in cutaneous T-cell lymphomas. Anticancer Res. 32, 1367–1373. 22493372

[B39] Newman M. (2010). Networks: An Introduction. Oxford: Oxford University Press 10.1093/acprof:oso/9780199206650.001.0001

[B40] Newman M. E. (2006). Modularity and community structure in networks. Proc. Natl. Acad. Sci. U.S.A. 103, 8577–8582. 10.1073/pnas.0601602103 16723398 PMC1482622

[B41] Odrowaz Z. Sharrocks A. D. (2012). The ETS transcription factors ELK1 and GABPA regulate different gene networks to control MCF10A breast epithelial cell migration. PLoS ONE 7:e49892. 10.1371/journal.pone.0049892 23284628 PMC3527487

[B42] Olson E. N. (2006). Gene regulatory networks in the evolution and development of the heart. Science 313, 1922–1927. 10.1126/science.1132292 17008524 PMC4459601

[B43] Palla G. Derényi I. Farkas I. Vicsek T. (2005). Uncovering the overlapping community structure of complex networks in nature and society. Nature 435, 814–818. 10.1038/nature03607 15944704

[B44] Potthoff M. J. Olson E. N. (2007). MEF2: a central regulator of diverse developmental programs. Development 134, 4131–4140. 10.1242/dev.008367 17959722

[B45] Power J. D. Cohen A. L. Nelson S. M. Wig G. S. Barnes K. A. Church J. A.. (2011). Functional network organization of the human brain. Neuron 72, 665–678. 10.1016/j.neuron.2011.09.006 22099467 PMC3222858

[B46] Ravasi T. Suzuki H. Cannistraci C. V. Katayama S. Bajic V. B. Tan K.. (2010). An atlas of combinatorial transcriptional regulation in mouse and man. Cell 140, 744–752. 10.1016/j.cell.2010.01.044 20211142 PMC2836267

[B47] Rives A. W. Galitski T. (2003). Modular organization of cellular networks. Proc. Natl. Acad. Sci. U.S.A. 100, 1128–1133. 10.1073/pnas.0237338100 12538875 PMC298738

[B48] Rosvall M. Bergstrom C. T. (2007). An information-theoretic framework for resolving community structure in complex networks. Proc. Natl. Acad. Sci. U.S.A. 104, 7327–7331. 10.1073/pnas.0611034104 17452639 PMC1855072

[B49] Rosvall M. Bergstrom C. T. (2008). Maps of random walks on complex networks reveal community structure. Proc. Natl. Acad. Sci. U.S.A. 105, 1118–1123. 10.1073/pnas.0706851105 18216267 PMC2234100

[B50] Sartorelli V. Huang J. Hamamori Y. Kedes L. (1997). Molecular mechanisms of myogenic coactivation by p300: direct interaction with the activation domain of MyoD and with the MADS box of MEF2C. Mol. Cell. Biol. 17, 1010–1026. 10.1128/MCB.17.2.1010 9001254 PMC231826

[B51] Sethi A. Eargle J. Black A. A. Luthey-Schulten Z. (2009). Dynamical networks in tRNA: protein complexes. Proc. Natl. Acad. Sci. U.S.A. 106, 6620–6625. 10.1073/pnas.0810961106 19351898 PMC2672494

[B52] Shannon C. E. Weaver W. (1959). The Mathematical Theory of Communication. Champaign, IL: University of Illinois Press.

[B53] Spirin V. Mirny L. A. (2003). Protein complexes and functional modules in molecular networks. Proc. Natl. Acad. Sci. U.S.A. 100, 12123–12128. 10.1073/pnas.2032324100 14517352 PMC218723

[B54] Suzuki H. Forrest A. R. van Nimwegen E. Daub C. O. Balwierz P. J. Irvine K. M.. (2009). The transcriptional network that controls growth arrest and differentiation in a human myeloid leukemia cell line. Nat. Genet. 41, 553–562. 10.1038/ng.375 19377474 PMC6711855

[B55] Tang B. Hsu H.-K. Hsu P.-Y. Bonneville R. Chen S.-S. Huang T. H.. (2012). Hierarchical modularity in ERα transcriptional network is associated with distinct functions and implicates clinical outcomes. Sci. Rep. 2:875. 10.1038/srep00875 23166858 PMC3500769

[B56] Taylor A. Halene S. (2015). The regulatory role of serum response factor pathway in neutrophil inflammatory response. Curr. Opin. Hematol. 22, 67–73. 10.1097/MOH.0000000000000099 25402621 PMC4374983

[B57] Thattai M. Van Oudenaarden A. (2001). Intrinsic noise in gene regulatory networks. Proc. Natl. Acad. Sci. U.S.A. 98, 8614–8619. 10.1073/pnas.151588598 11438714 PMC37484

[B58] Thevenon J. Bourredjem A. Faivre L. Cardot-Bauters C. Calender A. Murat A.. (2013). Higher risk of death among MEN1 patients with mutations in the JunD interacting domain: a Groupe detude des Tumeurs Endocrines (GTE) cohort study. Hum. Mol. Genet. 22, 1940–1948. 10.1093/hmg/ddt039 23376981

[B59] Tong Y. Merino D. Nimmervoll B. Gupta K. Wang Y.-D. Finkelstein D.. (2015). Cross-species genomics identifies TAF12, NFYC, and RAD54L as choroid plexus carcinoma oncogenes. Cancer Cell 27, 712–727. 10.1016/j.ccell.2015.04.005 25965574 PMC4458854

[B60] Vaughan C. A. Deb S. P. Deb S. Windle B. (2014). Preferred binding of gain-of-function mutant p53 to bidirectional promoters with coordinated binding of ETS1 and GABPA to multiple binding sites. Oncotarget 5, 417. 10.18632/oncotarget.1708 24481480 PMC3964217

[B61] Wang C.-C. Bajikar S. S. Jamal L. Atkins K. A. Janes K. A. (2014). A time-and matrix-dependent TGFBR3–JUND–KRT5 regulatory circuit in single breast epithelial cells and basal-like premalignancies. Nat. Cell Biol. 16, 345–356. 10.1038/ncb2930 24658685 PMC4035356

[B62] Wang J. Duncan D. Shi Z. Zhang B. (2013). WEB-based gene set analysis toolkit (WebGestalt): update 2013. Nucl. Acids Res. 41, W77–W83. 10.1093/nar/gkt439 23703215 PMC3692109

[B63] Wilkinson D. M. Huberman B. A. (2004). A method for finding communities of related genes. Proc. Natl. Acad. Sci. U.S.A. 101(Suppl. 1), 5241–5248. 10.1073/pnas.0307740100 14757821 PMC387302

[B64] Xie J. Kelley S. Szymanski B. K. (2013). Overlapping community detection in networks: the state-of-the-art and comparative study. ACM Comput. Surveys 45, 43 10.1145/2501654.2501657

[B65] Zerbini L. F. de Vasconcellos J. F. Czibere A. Wang Y. Paccez J. D. Gu X.. (2011). JunD-mediated repression of GADD45α and γ regulates escape from cell death in prostate cancer. Cell Cycle 10, 2583–2591. 10.4161/cc.10.15.16057 21734453 PMC3180197

[B66] Zhang B. Kirov S. Snoddy J. (2005). WebGestalt: an integrated system for exploring gene sets in various biological contexts. Nucl. Acids Res. 33(Suppl. 2), W741–W748. 10.1093/nar/gki475 15980575 PMC1160236

[B67] Zhu J. Zhang B. Smith E. N. Drees B. Brem R. B. Kruglyak L.. (2008). Integrating large-scale functional genomic data to dissect the complexity of yeast regulatory networks. Nat. Genet. 40, 854–861. 10.1038/ng.167 18552845 PMC2573859

[B68] Zou T. Rao J. N. Liu L. Xiao L. Chung H. K. Li Y.. (2015). JunD enhances miR-29b levels transcriptionally and posttranscriptionally to inhibit proliferation of intestinal epithelial cells. Am. J. Physiol. Cell Physiol. 308, C813–C824. 10.1152/ajpcell.00027.2015 25788572 PMC4436990

